# Diffusion-Based Sound Source Localization Using a Distributed Network of Microphone Arrays

**DOI:** 10.3390/s25072078

**Published:** 2025-03-26

**Authors:** Davide Albertini, Alberto Bernardini, Gioele Greco, Augusto Sarti

**Affiliations:** Dipartimento di Elettronica, Informazione e Bioingegneria, Politecnico di Milano, 20133 Milan, Italy; davide.albertini@polimi.it (D.A.); gioele.greco@polimi.it (G.G.); augusto.sarti@polimi.it (A.S.)

**Keywords:** sound source localization, wireless acoustic sensor networks, ATC diffusion, microphone array processing

## Abstract

Traditionally, microphone array networks for 3D sound source localization rely on centralized data processing, which can limit scalability and robustness. In this article, we recast the task of sound source localization (SSL) with networks of acoustic arrays as a distributed optimization problem. We then present two resolution approaches of such a problem; one is computationally centralized, while the other is computationally distributed and based on an Adapt-Then-Combine (ATC) diffusion strategy. In particular, we address 3D SSL with a network of linear microphone arrays, each of which estimates a stream of 2D directions of arrival (DoAs) and they cooperate with each other to localize a single sound source. We develop adaptive cooperation strategies to penalize the arrays with the most detrimental effects on localization accuracy and improve performance through error-based and distance-based penalties. The performance of the method is evaluated using increasingly complex DoA stream models and simulated acoustic environments characterized by various levels of reverberation and signal-to-noise ratio (SNR). Furthermore, we investigate how the performance is related to the connectivity of the network and show that the proposed approach maintains high localization accuracy and stability even in sparsely connected networks.

## 1. Introduction

In recent years, networks of distributed microphone arrays have gained popularity and have been used in various acoustic signal processing applications [1,2,3,4,5,6,7,8,9]. One of the most important tasks of these networks is sound source localization (SSL) and tracking, which can be a primary task or support other algorithms for which the position of one or more sound sources is valuable information [10,11,12,13,14,15,16,17]. Typical application scenarios for SSL methods are audio surveillance, video conferencing, and automotive systems [3,18,19].

The SSL problem has been extensively studied in the literature on distributed microphone arrays, and several approaches can be classified based on the type of *acoustic parameter* used for localization [1]. Examples of acoustic parameters used for SSL are time delay between microphones [10,11,12,16,20], measurements of sound energy [21,22], power measures obtained through beamforming techniques [13,17,23], and estimation of the direction of arrival (DoA) of sound sources [14,24,25,26,27,28,29]. More recently, techniques based on deep learning have used latent space features to map acoustic signals to the position of sound sources [15].

In this manuscript, we focus on SSL methods that utilize DoAs, which are defined as either one angle (in 2D space) or two angles (in 3D space), indicating the direction of a sound source with respect to a reference direction. In particular, we focus on the SSL framework introduced in [24], which allows 3D SSL using only 2D DoA measurements. This approach is advantageous because 2D DoAs can be computed quite efficiently, and are therefore well suited for low-cost, low-power microphone arrays commonly used in microphone array networks.

Regardless of the chosen acoustic parameters for SSL, most methods are based on optimization problems, where the goal is to fit a sound propagation model with acoustic measurements or features extracted from acoustic arrays [1]. Traditionally, SSL methods use centralized processing to solve the aforementioned optimization problems, where data from all arrays in the network are collected and processed by a dedicated node, often referred to as the Fusion Center, which then performs localization [1]. As a result, systems of this kind have a critical point of failure (i.e., the Fusion Center) that must also guarantee a communication bandwidth high enough to process measurements from all sensor nodes [3]. Therefore, there is a growing interest in developing computationally distributed solutions that allow for estimation of the quantities of interest (e.g., the position of a sound source) by distributing the computational load to all acoustic arrays and leveraging their cooperation to achieve better performance. In addition, distributed approaches are desirable due to their higher scalability, robustness, and low power consumption [30].

Computationally distributed approaches in microphone array networks have been investigated for various acoustic signal processing tasks, including signal estimation [31,32], beamforming techniques [5,33], active noise control [34], and acoustic echo cancellation [35]. However, to the best of our knowledge, few computationally distributed SSL methods have been proposed, and they are mostly limited to 2D environments [17,36,37].

Recently, the authors of the present manuscript, building on the centralized framework for 3D SSL with 2D DoAs of [24], proposed a computationally distributed 3D SSL method [38] that uses a network of planar microphone arrays. In [38], SSL is described as a distributed minimization problem and is approached with an Adapt-Then-Combine (ATC) diffusion strategy [30,39]. This, in turn, has two major advantages over [24]. Firstly, the approach is computationally distributed and divides the computational load across the arrays. Second, the approach is adaptive, i.e., each array handles a stream of 2D DoAs instead of single DoA measurements as in [24]. This allows the microphone array network to adapt to changes in the distribution of DoA streams, which are usually influenced by noise and unfavorable acoustic effects, and learn their statistical moments. As a result, localization accuracy can be improved by penalizing arrays that acquire unreliable measurements. By responding to DoA streams, the tracking of sound sources is also automatically integrated into this approach, under the assumption that the sound source moves relatively slowly.

In this manuscript, we extend the work of the conference paper [38] in several ways. With respect to [38], which considers a simple DoA stream model based on the assumption that the acoustic environment is anechoic, this manuscript introduces more sophisticated DoA stream models, along with applications of the method in simulated reverberant environments. Building on these extensions, we propose a new set of data exchange policies between acoustic arrays that are specifically tailored to significantly improve localization accuracy in more complex acoustic scenarios. Moreover, the work in [38] considered just a *fully connected* network topology, where each node is directly connected to every other node. In this manuscript, we consider different connected topologies and investigate how performance is affected when network connectivity is gradually reduced. Our results show that the performance degradation is negligible as long as a connected network topology is considered. This emphasizes the effectiveness and resilience of the proposed distributed approach. We also test the robustness of the proposed approach at different reverberation levels and signal-to-noise ratios (SNRs). We show that our method converges even under challenging conditions and show a way to control the stability of the position estimate after convergence.

The manuscript is structured as follows. Section 2 introduces the SSL framework used throughout the paper and formulates the SSL task as an optimization problem. Section 3 discusses a centralized solution to this optimization problem that is able to handle streams of DoAs and penalize noisy arrays. Section 4 presents a computationally distributed approach that uses an ATC diffusion strategy to solve the SSL problem. This section also introduces new cooperation strategies between array nodes to improve performance. Section 5 evaluates the accuracy and robustness of the proposed methods, while Section 6 examines the convergence speed and steady-state stability of the approach. Section 7 assesses the resilience of the method under reduced network connectivity. Finally, Section 8 offers concluding remarks and discusses potential future developments.

## 2. Background on 3D Sound Source Localization with Linear Microphone Arrays

We tackle the problem of localizing a sound source in a 3D space using a network of linear microphone arrays, where each array measures a 2D DoA. Let us consider an acoustic environment in which a single sound source is located at the coordinates $s={\left[s_{x},s_{y},s_{z}\right]}^{T}$. Let us also consider *K* spatially distributed linear microphone arrays whose reference points are located at the coordinates $m_{k}={\left[m_{k,x},m_{k,y},m_{k,z}\right]}^{T}$ for k=1,…,K. Each array is oriented according to a unit vector $v_{k}={\left[\cos\alpha_{k}\cos\beta_{k},\sin\alpha_{k}\cos\beta_{k},\sin\beta_{k}\right]}^{T}$, where $\alpha_{k}$ and $\beta_{k}$ stand for the azimuth and elevation angle, respectively. The goal is to determine the position of the sound source based on the DoAs detected by the microphone arrays.

### 2.1. Sound Source Localization Framework

Similarly to the work in [24], in this work we approach 3D SSL with linear arrays by performing 2D DoA triangulation in the same plane as the sound source; namely, the plane defined by $z=s_{z}$. The goal of triangulation for SSL is to determine the location of the sound source as the intersection of acoustic rays emanating from the source and passing through the microphone arrays, where the acoustic rays are parameterized by the position of the array and its DoA estimate. Since in our context the triangulation takes place on the plane $z=s_{z}$, this poses a challenge as the acoustic rays from the source to the microphone arrays do not normally lie on this plane. Therefore, we consider the projection of the microphone arrays onto the plane $z=s_{z}$. The coordinates of the projected arrays’ reference points are denoted as $m_{k}^{\prime }={\left[m_{k,x}^{\prime },m_{k,y}^{\prime },s_{z}\right]}^{T}$. To ensure the correctness of this approach, each projected array must preserve the 2D DoA $\vartheta_{k}=\theta_{k}+\alpha_{k}$, where $\theta_{k}$ is the local DoA estimate of the array. The distance of the array to the sound source must also be preserved after projection, i.e., $||s-m_{k}\left|\right|=||s-m_{k}^{\prime }\left|\right|$. Given these constraints, the projection of each array depends on the sound source position s, and the coordinates are computed as [24]

(1) $$\begin{array}{cc} m_{k,x}^{\prime } & =s_{x}-C_{k}P_{k}+Q_{k}\sqrt{D_{k}-C_{k}^{2}},\\ m_{k,y}^{\prime } & =s_{y}-C_{k}Q_{k}-P_{k}\sqrt{D_{k}-C_{k}^{2}}, \end{array}$$

where

$$\begin{array}{c} \begin{array}{cccc} C_{k} & =v_{k}^{T}\left(s-m_{k}\right), & D_{k} & ={∥s-m_{k}∥}^{2},\\ P_{k} & =\cos\alpha_{k}, & Q_{k} & =\sin\alpha_{k}. \end{array} \end{array}$$

**Figure 1:**
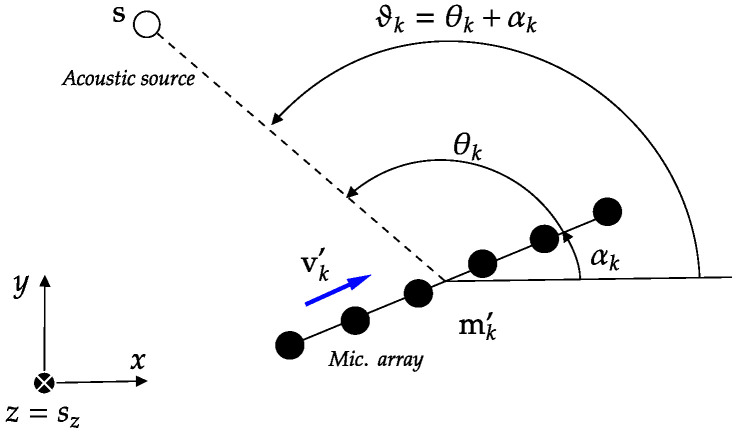
A projected microphone array centered at $m_{k}^{\prime }$, and orientation versor $v_{k}^{\prime }={\left[\cos\alpha_{k},\sin\alpha_{k},0\right]}^{T}$ measures the DoA $\theta_{k}$ of a sound source located at s in the far field.

A representation of a projected array and the corresponding local DoA is shown in Figure 1.

Having defined the parameters of the projected arrays, we can determine the acoustic rays required for triangulation on the plane $z=s_{z}$. An acoustic ray intersecting a sound source and a projected microphone array *k* is described by

(2) $$a_{k}s_{x}+b_{k}s_{y}+c_{k}=0,$$

where $a_{k}$, $b_{k}$, and $c_{k}$ are parameterized in terms of the DoA $\vartheta_{k}$ of the array as

(3) $$\left\{\begin{array}{c} a_{k}=\sin\left(\vartheta_{k}\right)\\ b_{k}=-\cos\left(\vartheta_{k}\right)\\ c_{k}=m_{k,y}^{\prime }\cos\left(\vartheta_{k}\right)-m_{k,x}^{\prime }\sin\left(\vartheta_{k}\right)\;. \end{array}\right.$$

However, in a real acoustic scenario, Equation (2) is generally not satisfied due to the error introduced by the DoA estimation process and adverse room acoustic phenomena. Nevertheless, from Equation (2), we can derive an error function that measures the agreement between the measurements of each array and the assumed acoustic ray propagation model. Therefore, we define the fitting error of each array DoA measure as

(4) $$e_{k}\left(s,\vartheta_{k}\right)=\sin\vartheta_{k}s_{x}-\cos\vartheta_{k}s_{y}+c_{k}\left(s,\vartheta_{k}\right).$$

Employing the fitting error of each array in (4), we can express the considered SSL task by writing the following optimization problem:

(5) $$\begin{array}{cc} \arg\min_{s}J^{\mathrm{glob}}\left(s\right) & =\arg\min_{s}\sum_{k=1}^{K}e_{k}{\left(s,\vartheta_{k}\right)}^{2}\\ \; & =\arg\min_{s}\sum_{k=1}^{K}J_{k}\left(s,\vartheta_{k}\right) \end{array}$$

where we use $J_{k}\left(s,\vartheta_{k}\right)=e_{k}{\left(s,\vartheta_{k}\right)}^{2}$ to denote the local cost function of array *k*. Consequently, by minimizing the global cost $J_{\mathrm{glob}}\left(s\right)$, we aim to find the source location s that best fits all measured DoAs across the network.

### 2.2. Centralized Resolution Approach Presented in [24]

A centralized solution to the optimization problem (5) can be obtained by using the following Gauss–Newton iterative method [24]:

(6) $$s_{i}=s_{i-1}-{\left[\nabla e(s_{i-1})\right]}^{†}e\left(s_{i-1}\right)$$

where $e\left(s_{i}\right)={\left[e_{1}\left(s_{i},\vartheta_{1}\right),e_{2}\left(s_{i},\vartheta_{2}\right),\ldots ,e_{K}\left(s_{i},\vartheta_{K}\right)\right]}^{T}$, $s_{i}={\left[s_{x,i},s_{y,i},s_{z,i}\right]}^{T}$ is the position estimate at iteration index *i*, ^†^ denotes the Moore–Penrose matrix inverse operator, and $\nabla e\left(s\right)={\left[\nabla e_{1}\left(s,\vartheta_{1}\right),\ldots ,\nabla e_{K}\left(s,\vartheta_{K}\right)\right]}^{T}$ is the error gradient vector with elements

$$\nabla e_{k}\left(s,\vartheta_{k}\right)={\left[\frac{\partial e_{k}\left(s,\vartheta_{k}\right)}{\partial s_{x}},\frac{\partial e_{k}\left(s,\vartheta_{k}\right)}{\partial s_{y}},\frac{\partial e_{k}\left(s,\vartheta_{k}\right)}{\partial s_{z}}\right]}^{T}.$$

Although the approach converges to a solution quite quickly [24], it has several limitations in terms of flexibility and localization performance. Regarding flexibility, Ref. [24] is designed for single DoA measurements and remains an “instantaneous” approach that is not able to learn from data streams and to adapt accordingly. Moreover, since it is computationally centralized, its scalability and robustness are limited. As far as localization performance is concerned, Ref. [24] lacks a mechanism to scale the contribution of individual array measurements, preventing the penalization of arrays that negatively impact localization accuracy.

### 2.3. Distributed Resolution Approach Presented in [38]

Our previous conference paper [38] presents a computationally distributed approach based on ATC diffusion. It is designed starting from the theoretical framework established in [24], adapting it to DoA streams while incorporating a mechanism to penalize arrays with the most detrimental effects on localization. However, Ref. [38] is also characterized by important limitations. Firstly, only anechoic environments were considered for the performance evaluation, resulting in a simple DoA stream model. Moreover, only fully connected topologies were considered and no studies on the performance of networks with reduced connectivity were presented.

## 3. Adaptive Sound Source Localization

We first present a computationally centralized approach to solving the 3D SSL problem outlined in Section 2. This method improves upon the one proposed in [24] by incorporating penalty factors to scale down the contribution of arrays that negatively impact the localization task. In preparation for this discussion, we briefly describe the network model.

### 3.1. Network Topology

**Figure 2:**
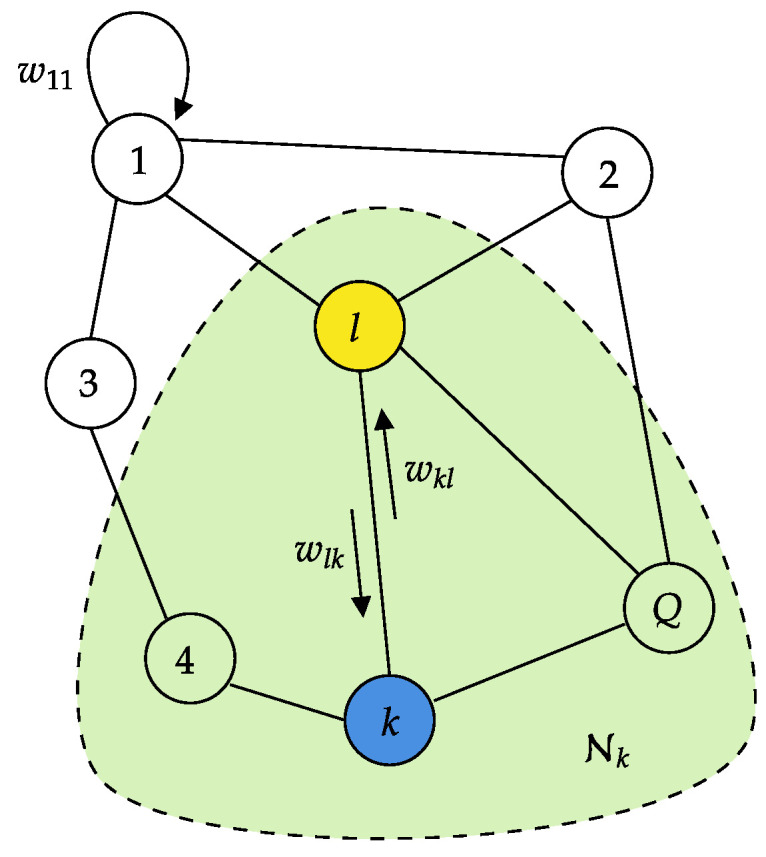
Example of network topology with *K* microphone arrays. The green area highlights the neighborhood $N_{k}$ of agent *k*.

Let us assume the *K* microphone arrays are connected by a network topology modeled as a digraph, where the nodes represent the microphone arrays and the edges represent communication links between the node pairs. We define the neighborhood of a node *k* as the set $N_{k}$ of all nodes connected to *k* by an edge. Given two neighboring nodes *k* and *l*, a non-negative scalar $w_{lk}$ is used by node *k* to scale the data it receives from node *l*, and can be interpreted as the degree of trust node *k* assigns to node *l*, and the converse holds for $w_{kl}$. A depiction of a network of this sort is shown in Figure 2. We can collect the scaling coefficients of the entire network into an K×K *combination matrix* $W=[w_{lk}]$.

To enforce convergence and stability properties, it is common to require W to be a left-stochastic and primitive matrix [39,40]. Left-stochasticity is enforced by requiring all columns of W to sum to one. Additionally, we impose a strongly connected topology, ensuring a path exists between any two nodes and that at least one node is connected to itself. This last condition automatically guarantees that W is primitive [30].

### 3.2. Centralized Adaptive SSL

A first step towards a more general SSL problem formulation of (5), which enables the introduction of penalty factors to scale the contribution of noisy arrays, is given by

(7) $$\arg\min_{s}J^{\mathrm{weight}}\left(s\right)=\arg\min_{s}\sum_{k=1}^{K}q_{k}J_{k}\left(s,\vartheta_{k}\right).$$

This more general problem formulation leads to different solutions compared to the unweighted problem in (5). The solutions of these two optimization problems coincide only if the $q_{k}$ terms are equal or if the local costs are minimized at the same location [30]. However, in the SSL framework under consideration, this is not the case. From the theory of adaptive networks, we know that the solution of (7) can be interpreted as a Pareto-optimal solution [40]. Different choices of the scaling factors $q_{k}$ will lead to different Pareto-optimal solutions. From now on, we denote a Pareto-optimal solution with $s^{*}$, which we also call the *network’s limit point*. It is important to emphasize that the limit point $s^{*}$ does not necessarily correspond to the actual position of the source, since in real scenarios biases of DoA estimations lead to different solutions.

For the solution of (7), we here consider a centralized approach based on stochastic gradient descent (SGD) and characterized by the following iteration:

(8) $$s_{i}=s_{i-1}-\sum_{k=1}^{K}q_{k}\nabla_{s}J_{k}\left(s_{i-1},\vartheta_{k,i}\right)$$

where $\nabla_{s}$ denotes the gradient operator with respect to s. Unlike in Equation (6), here the instantaneous DoA measurements at each node are replaced by DoA streams, i.e., $\vartheta_{k}=\vartheta_{k,i}$, which endows the approach with learning and adaptation capabilities. Note also that the use of DoA streams leads to perturbations during descent to a Pareto-optimal solution $s^{*}$. These perturbations are inherent in SGD-based approaches, as the local costs and thus the global cost function are evaluated with noisy DoA measures. As a result, the network never fully reaches $s^{*}$, but continues to orbit around this solution after an initial transient.

Without loss of generality, we express $q_{k}$ as $q_{k}=\mu_{k}p_{k}\left(W\right)$, where $\mu_{k}$ is an array-specific parameter, while $p_{k}\left(W\right)$ depends on the network topology. This leads to the following centralized approach:

(9) $$s_{i}=s_{i-1}-\sum_{k=1}^{K}\mu_{k}p_{k}\left(W\right)\nabla_{s}J_{k}\left(s_{i-1},\vartheta_{k,i}\right).$$

Here, $\mu_{k}>0$ is a step-size parameter, and is used to determine the adaptation speed for each array. Instead, $p_{k}\left(W\right)$ is set as the *k*th entry of the Perron eigenvector of the combination matrix W. Since we constrain W to be left-stochastic and primitive, the existence of the Perron eigenvector is guaranteed by the Perron–Frobenius theorem [41]. More precisely, the eigenvector satisfies

(10) $$\mathrm{Wp}=p,\;1^{T}p=1,\;p_{k}>0,\;k=1,\ldots ,K.$$

This specific choice of $p_{k}\left(W\right)$ aligns with the goal of obtaining the equivalent centralized implementation of the distributed diffusion ATC approach described in [38], which considers only fully connected networks. Specifically, this choice ensures that the centralized SGD approach of (9) yields the same iterates $s_{i}$ of the corresponding fully connected distributed implementation, having as its combination matrix W [39]. The process of setting the combination weights in W in order to scale noisy arrays is described in Section 4.2.

## 4. ATC Diffusion Sound Source Localization

In this section, we extend the computationally distributed approach of [38] to solve the problem in (7). First, we revisit the ATC diffusion-based 3D SSL approach of [38]. Then, we introduce novel cooperation strategies and propose two metrics to evaluate distributed approaches for SSL.

### 4.1. ATC SSL Algorithm

According to [38], a computationally distributed solution to (7) can be found by employing a distributed version of the SGD, called *Adapt-Then-Combine (ATC) diffusion* [30]. The ATC diffusion method consists of two steps. In the *adapt* step, a local SGD step is performed by each node. In the *combine* step, each node combines the results of its neighbors. Applying ATC diffusion to the problem in Equation (7) leads to the following recursion, defined for each agent *k*:

(11) $$\left\{\begin{array}{c} \psi_{k,i}=s_{k,i-1}-\mu_{k}\nabla_{s}J_{k}\left(s_{k,i-1},\vartheta_{k,i}\right)\\ s_{k,i}=\sum_{l\in N_{k}}w_{lk}\psi_{l,i} \end{array}\right..$$

As each local cost function in (5) is a quadratic term, its gradient can be expressed as

(12) $$\nabla_{s}J_{k}\left(s,\vartheta_{k}\right)=2e_{k}\left(s,\vartheta_{k}\right)\nabla_{s}e_{k}\left(s,\vartheta_{k}\right).$$

Therefore, we can rewrite (11) as

(13) $$\left\{\begin{array}{c} \psi_{k,i}=s_{k,i-1}-2\mu_{k}e_{k}\left(s_{k,i-1},\vartheta_{k,i}\right)\nabla_{s}e_{k}\left(s_{k,i-1},\vartheta_{k,i}\right)\\ s_{k,i}=\sum_{l\in N_{k}}w_{lk,i}\psi_{l,i} \end{array}\right.$$

where

(14) $$\nabla_{s}e_{k}\left(s,\vartheta_{k}\right)=\left[\begin{array}{c} -a_{k}\left(Q_{k}\Theta_{k,x}-v_{k,x}P_{k}\right)+b_{k}\left(P_{k}\Theta_{k,x}+v_{k,x}Q_{k}\right)\\ -a_{k}\left(Q_{k}\Theta_{k,y}-v_{k,y}P_{k}\right)+b_{k}\left(P_{k}\Theta_{k,y}+v_{k,y}Q_{k}\right)\\ -a_{k}\left(Q_{k}\Theta_{k,z}-v_{k,z}P_{k}\right)+b_{k}\left(P_{k}\Theta_{k,z}+v_{k,z}Q_{k}\right) \end{array}\right]$$

and

(15) $$\Theta_{k,j}=\frac{s_{j}-m_{k,j}-v_{k,j}C_{k}}{\sqrt{D_{k}-C_{k}^{2}}}\;\mathrm{with}\;j=x,y,z.$$

It is worth noting that the quantities $a_{k}$ and $b_{k}$ depend on the instantaneous DoA measures $\vartheta_{k,i}$, while the quantities $C_{k}$ and $D_{k}$ depend on the previous array position estimate $s_{k,i-1}$, but such dependencies have been omitted for the sake of readability.

From Equation (13), we also note that there are two sets of tunable parameters that can influence the dynamics of the ATC diffusion recursion, namely, the step-size parameter $\mu_{k}$ and the combination weights $w_{lk}$. Both parameters influence the solution of the problem in Equation (7), leading to different Pareto-optimal solutions $s^{*}$ [40]. From this point on, we assume a uniform step-size parameter $\mu_{k}=\mu$ across all nodes, so that it no longer influences the attained solutions $s^{*}$. Instead, combination weights $w_{lk}$ are set to achieve different limit point solutions $s^{*}$ [30,39], and we refer to the policies for determining these coefficients as *combination policies*. In the context of SSL, it is desirable to adjust these coefficients to obtain limit point solutions $s^{*}$ that are closer to the actual sound source, thereby increasing localization accuracy.

In the case where the network is fully connected, and thus no non-zero weights are present in the combination matrix W, it is worth noting that each node could potentially compute the centralized implementation discussed in Section 3.2. This implementation is characterized by the same localization accuracy and convergence behavior as the distributed, fully connected implementation.

### 4.2. Adaptive Combination Policies

We now discuss how combination policies can be used to improve the localization accuracy of the proposed distributed SSL method. In general, we can distinguish two classes of combination policies: one where the combination coefficients are kept constant and one where they change from iteration to iteration [30]. In this paper, we focus on the latter case and thus refer to the resulting combination strategies as *adaptive* combination strategies. The main idea is to adaptively adjust the combination weights in the matrix W to penalize arrays that have the most detrimental effect on localization accuracy by assigning them small combination weights. To achieve this goal, at each iteration *i*, each agent *k* assigns a penalty term $\gamma_{l,i}$, with $l\in N_{k}$, to its neighbors as

(16) $$\gamma_{l,i}=\left(1-\zeta \right)\gamma_{l,i-1}+\zeta \varphi_{l,i},$$

where $\varphi_{l,i}$ is an arbitrary penalty factor for the current iteration and 0≤ζ≤1 is a forget rate that smooths the penalty factors. Then, the combination weights assigned by node *k* to its neighbors are calculated as follows:

(17) $$w_{lk,i}=\left\{\begin{array}{c} \frac{1}{\gamma_{l,i}}{\left(\sum_{l\in N_{k}}\frac{1}{\gamma_{l,i}}\right)}^{-1},\;\mathrm{if}\;l\in N_{k}\\ 0,\;\mathrm{otherwise} \end{array}\right.$$

where we normalize the weights so that the resulting combination matrix is left-stochastic. Penalty factors can be chosen in several ways. Below, we propose three different combination policies aimed at increasing the localization accuracy of ATC diffusion SSL and discuss their applicability.

#### 4.2.1. Error Penalty Factor

The first penalty factor we propose utilizes the fitting errors of neighboring nodes. At each iteration, each agent *k* penalizes its neighbors based on $\left|e_{l}\left(s_{l,i-1},\vartheta_{l,i}\right)\right|$, where $l\in N_{k}$. This penalty factor represents the absolute value of the fitting error between the source position estimate and the data measurements from the neighboring nodes. Thus, the penalty factor is calculated as follows:

(18) $$\varphi_{l,i}=\left|e_{l}\left(s_{l,i-1},\vartheta_{l,i}\right)\right|,$$

and then smoothed according to Equation (16).

#### 4.2.2. Cost Penalty Factor

Another option is to utilize the values of the cost functions associated with the neighbors of each node as a penalty factor. As the cost function is defined as a quadratic error, each agent can employ the following penalty factor for its neighbors:

(19) $$\varphi_{l,i}=J_{l}\left(s_{l,i-1},\vartheta_{l,i}\right)=e_{l}^{2}\left(s_{l,i-1},\vartheta_{l,i}\right).$$

Furthermore, assuming a uniform step size among all nodes, i.e., $\mu_{l}=\mu_{k}=\mu$, and a fully connected network topology, it is possible to verify that the policy resulting from the employment of this penalty factor corresponds to the well-known neighbor-centered *adaptive relative variance* combination policy [30] from adaptive networks theory.

#### 4.2.3. Distance Penalty Factor

As a last option, we propose to use the distance between each array and the predicted position of the sound source as the penalty factor. This is because, especially in reverberant environments, distant sources are expected to impair the DoA estimation methods more than nearby sources. Furthermore, distant arrays impair the localization more than nearby arrays for the same DoA error. Therefore, we may use the following penalty factor:

(20) $$\varphi_{l,i}={∥m_{l}-s_{l,i-1}∥}^{2}\;.$$

Regardless of which approach is used to determine the combination weights, each of the proposed policies requires the exchange of additional parameters between agents. Without any combination policies, nodes only exchange their estimates for the position of the sound source during the combine step of the ATC diffusion iteration. Similarly, the distance penalty does not require any additional data exchange if we assume that self-localization is performed once before SSL, allowing each node to know the positions of all its neighbors. Instead, when using either the error penalty in Equation (18) or the cost penalty in Equation (19), an additional parameter, namely, $e_{l}\left(s_{l,i-1},\vartheta_{l,i}\right)$, must be exchanged between neighboring nodes. However, even if the additional parameter $e_{l}\left(s_{l,i-1},\vartheta_{l,i}\right)$ is exchanged, the communication bandwidth of each agent remains small unless data are exchanged very frequently.

**Figure 3:**
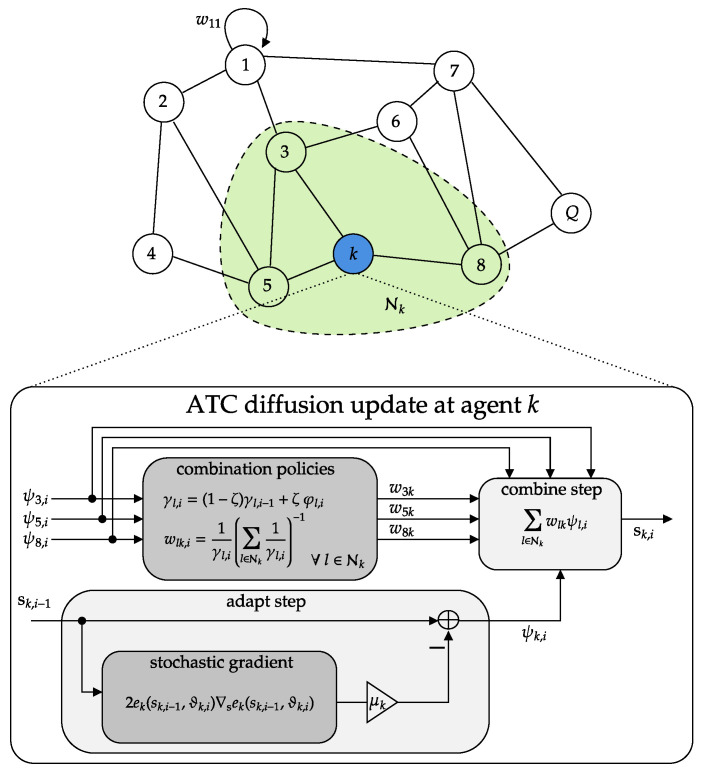
Schematic of the ATC diffusion-based SSL for node *k* in an example topology. Each node updates its local estimate (adapt step) and then merges intermediate estimates from neighbors (combine step) using an adaptive combination policy.

To summarize the proposed approach, Algorithm 1 provides the pseudocode for each agent, detailing the iterative application of diffusion-based SSL and the combination policy. Complementing this, Figure 3 presents the corresponding block scheme.
**Algorithm 1** Diffusion-Based SSL With Linear Arrays%For each array, at each new iteration *i*, do%Adapt Step$\psi_{k,i}=s_{k,i-1}-2\mu_{k}e_{k}\left(s_{k,i-1},\vartheta_{k,i}\right)\nabla_{s}e_{k}\left(s_{k,i-1},\vartheta_{k,i}\right)$%Select Combination Strategy**for** each neighbor $l\in N_{k}$ **do**   %ComputePenaltyFactor $\varphi_{l,i}$ accordingto(18)–(20)   $\gamma_{l,i}=\left(1-\zeta \right)\gamma_{l,i-1}+\zeta \varphi_{l,i}$   $w_{lk,i}=\frac{1}{\gamma_{l,i}}{\left(\sum_{l\in N_{k}}\frac{1}{\gamma_{l,i}}\right)}^{-1},\;\mathrm{if}\;l\in N_{k}$**end for**%Combine Step$s_{k,i}=\sum_{l\in N_{k}}w_{lk,i}\psi_{l,i}$

### 4.3. Metrics

To evaluate the performance of distributed SSL and the effectiveness of the different combination strategies, we introduce two metrics, namely, the mean absolute error (MAE) and the mean squared deviation (MSD). Both metrics are used to describe the steady-state behavior of the network and are calculated for each sensor node. An averaged version of these metrics across all nodes can be used to evaluate the global performance of the network.

#### 4.3.1. Mean Absolute Error

The MAE evaluates localization accuracy by evaluating the error between the predicted source position and the actual position of the sound source, which we define as $s^{\mathrm{GT}}$. Mathematically, we define the node and the network MAE as

(21) $$\begin{array}{cc} {\mathrm{MAE}}_{k} & ≜\lim_{i\to \infty }E∥s_{k,i}-s^{\mathrm{GT}}∥,\\ {\mathrm{MAE}}_{\mathrm{avg}} & ≜\frac{1}{K}\sum_{k=1}^{K}{\mathrm{MAE}}_{k}\;, \end{array}$$

respectively. In practice, we cannot use the theoretical definitions of the MAE as in Equation (21) because these require averaging over an infinite number of iterations. Therefore, in this paper, we estimate the MAE by stopping the diffusion-based SSL algorithm after $I_{\max}>>1$ iterations to ensure that the network has reached a steady state and estimate the node and network MAE as

(22) $$\begin{array}{cc} {\hat{\mathrm{MAE}}}_{k} & ≜\frac{1}{N_{\mathrm{it}}}\sum_{i=I_{\max}-N_{\mathrm{it}}}^{I_{\max}}∥s_{k,i}-s^{\mathrm{GT}}∥\\ {\hat{\mathrm{MAE}}}_{\mathrm{avg}} & ≜\frac{1}{K}\sum_{k=1}^{K}{\hat{\mathrm{MAE}}}_{k}\; \end{array}$$

where $N_{\mathrm{it}}$ denotes the number of steady-state iterations.

#### 4.3.2. Mean Square Deviation

MSD evaluates the stability properties of diffusion methods [30] by quantifying the mean squared error (MSE) between each node’s iteration at steady state and the estimated network limit point. We define the MSD of each node and that of the entire network as

(23) $$\begin{array}{cc} {\mathrm{MSD}}_{k} & ≜\lim_{i\to \infty }E∥s_{k,i}-s^{*}∥,\\ {\mathrm{MSD}}_{\mathrm{avg}} & ≜\frac{1}{K}\sum_{k=1}^{K}{\mathrm{MSD}}_{k}\;. \end{array}$$

Similarly to before, we cannot use the theoretical definitions of MSD as in Equation (21) because we do not know the limit point of the network $s^{*}$ and the definitions are averages over an infinite number of iterations. Moreover, it is often more interesting to show “instantaneous” values of the MSD, which show how the deviations of the iterates around the reached network limit points evolve over time. In this sense, we define the “instantaneous” MSD as

(24) $$\begin{array}{cc} {\hat{\mathrm{MSD}}}_{k}\left(i\right) & ≜∥s_{k,i}-{\hat{s}}^{*}∥\\ {\hat{\mathrm{MSD}}}_{\mathrm{avg}}\left(i\right) & ≜\frac{1}{K}\sum_{k=1}^{K}{\hat{\mathrm{MSD}}}_{k}\;. \end{array}$$

where the network limit point is estimated as

(25) $${\hat{s}}^{*}≜\frac{1}{KN_{\mathrm{it}}}\sum_{k=1}^{K}\sum_{i=I_{\max}-N_{\mathrm{it}}}^{I_{\max}}s_{k,i}.$$

## 5. Testing the Localization Accuracy

In this section, we evaluate the localization accuracy of the proposed diffusion-based SSL method using both DoA stream models and an acoustic simulation. The evaluation is performed under a fully connected network topology, establishing a clear baseline for assessing the impact of combination policies without the complexities introduced by sparser connectivity. The influence of partial network connectivity is explored in Section 7. Throughout this section, we consider a room of size 4m×3m×3m, with eight microphone arrays placed near the room edges, with reference positions $m_{k}$ and orientations $v_{k}$ set according to Table 1. We also consider different candidate sound source positions $s^{\mathrm{GT}}$ placed on a uniformly distributed 2D grid of 23×23 points for two different planes: z=0.45 and z=1.5. As for the diffusion-based SSL method, we consider a uniform step size across all nodes $\mu_{k}=\mu =0.1$. Sound sources are assumed to be stationary in this set of experiments.

### 5.1. DoA Stream Models

We begin by evaluating the accuracy of the proposed ATC diffusion SSL method using different DoA stream models. These models represent the combined effects that room acoustics and the DoA estimators used in each array have on the generation of DoA streams. This approach allows us to test the accuracy of the method in controlled, simplified scenarios and to investigate how localization accuracy is affected by factors such as biases in DoA estimation.

We employ three different DoA stream models. Each of them is a perturbed version of the actual DoA $\theta_{k}^{\mathrm{GT}}$, which we define as

(26) $$\theta_{k}^{\mathrm{GT}}=\arccos\left(\frac{v_{k}^{T}\left(s^{\mathrm{GT}}-m_{k}\right)}{∥s^{\mathrm{GT}}-m_{k}∥}\right).$$

The simplest DoA stream model is based on the assumption that DoAs measured by each array are drawn from the following distribution:

(27) $$\theta_{k,i}∼G\left(\theta_{k}^{\mathrm{GT}},\sigma \right),$$

where G(ν,ϖ) is a normal distribution with mean ν and variance ϖ. Hence, it is assumed that each agent measures unbiased DoAs with the same variance σ. We will refer to this DoA measurement distribution as *model I*. This was the only case considered in [38].

A more complex DoA stream model also considers a different DoA measurement variance for each array, i.e., DoAs are generated from the distribution

(28) $$\theta_{k,i}∼G\left(\theta_{k}^{\mathrm{GT}},\sigma_{k}\right).$$

We refer to this DoA stream distribution as *model II*, according to which each agent has its own variance $\sigma_{k}∼L\left(\nu ,\varpi \right)$, and L denotes a log-normal distribution with mean ν and variance ϖ.

However, in real acoustic scenarios DoA estimates are often biased, due to reverberation and a low SNR. Therefore, a more realistic DoA stream model, which we refer to as *model III*, considers biased agents, each with its own measurement bias. The distribution for model III is given by

(29) $$\theta_{k,i}∼G\left(\nu_{k},\sigma_{k}\right),$$

where $\nu_{k}∼G\left(\theta_{k}^{\mathrm{GT}},\sigma_{b}\right)$ and $\sigma_{b}$ models the variance of the measurement bias, and where $\sigma_{k}$ has the same distribution as model II.

We now compare the localization accuracy of the proposed diffusion-based SSL with the various DoA stream models discussed above. In particular, we evaluate the accuracy by computing the localization error ${\hat{\;\mathrm{MAE}}}_{\mathrm{avg}}$ according to Equation (22) and we perform $N_{\mathrm{trials}}$ Monte Carlo simulations for each possible source position in the grid to average the effect of the different DoA stream realizations.

**Figure 4:**
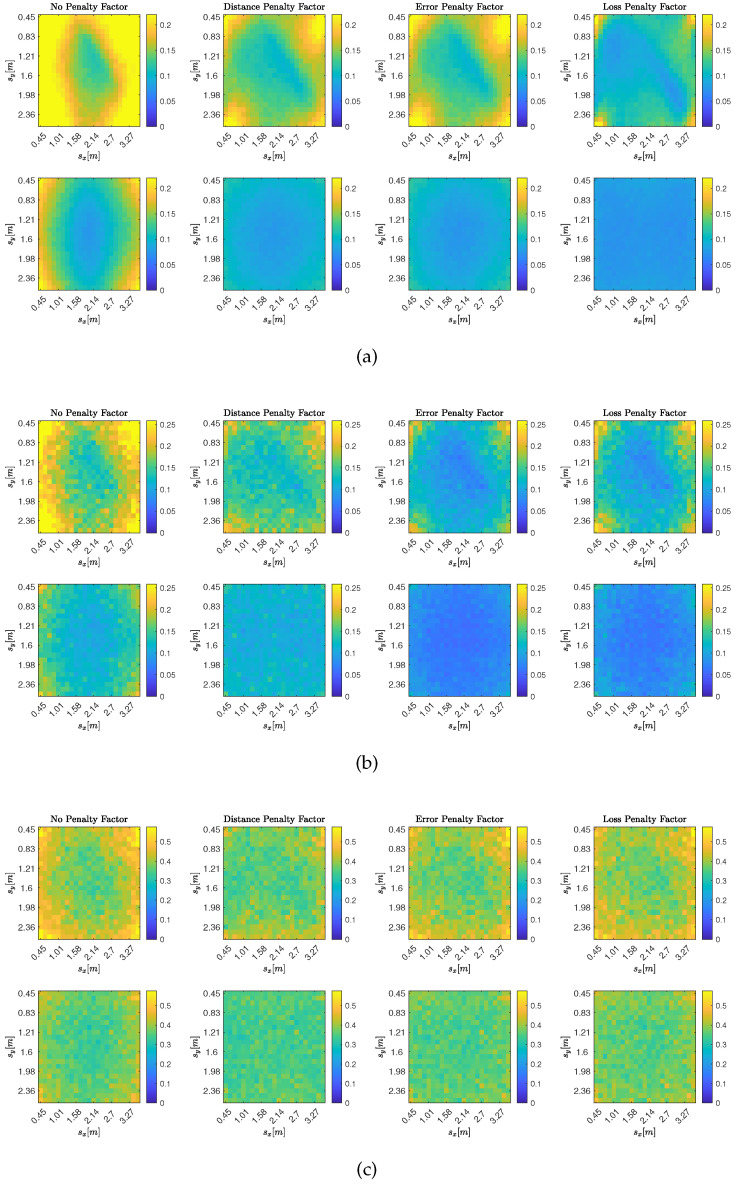
Average MAE, ${\hat{\mathrm{MAE}}}_{\mathrm{avg}}$, for the DoA stream models from Section 5.1. Each subfigure shows results on a 23×23 grid in the xy-plane at z=0.45 (top) and z=1.5 (bottom), with $N_{\mathrm{trials}}=50$ realizations per DoA stream model. Columns (from left to right) compare no combination strategy, distance combination strategy (20), error combination strategy (18), and cost combination strategy (19). (**a**) ${\hat{\mathrm{MAE}}}_{\mathrm{avg}}$ for DoA stream Model I with σ=10°. (**b**) ${\hat{\mathrm{MAE}}}_{\mathrm{avg}}$ for DoA stream Model II with $\sigma_{k}∼L\left(1.9^{{}^{\circ}},0.66{}^{\circ}\right)$. (**c**) ${\hat{\mathrm{MAE}}}_{\mathrm{avg}}$ for DoA stream Model III with $\sigma_{k}∼L\left(1.9{}^{\circ},0.6{}^{\circ}\right)$ and $\nu_{k}∼G\left(\theta_{k}^{\mathrm{GT}},0.8\right)$.

The results are shown in Figure 4, where the metric ${\hat{\mathrm{MAE}}}_{\mathrm{avg}}$ is shown at each possible source position for all different DoA stream models. In particular, top to bottom, Figure 4 depicts the accuracy of the proposed approach using DoA streams drawn from models I, II, and III, while different columns show different combination policies. The leftmost column shows the results when no adaptive combination policy is used and therefore all combination coefficients are set to $w_{lk,i}=w_{lk}=1/Q$. The other three columns, in order from left to right, in Figure 4 show the results obtained when the combination coefficients are set according to the distance penalty factor in Equation (20), the error penalty factor in Equation (18), and the cost penalty factor in Equation (19), respectively. For this analysis, we set $N_{\mathrm{trials}}=50$, $N_{\mathrm{it}}=100$, and $I_{\max}=500$. These values were selected to ensure that, from iteration $I_{\max}-N_{\mathrm{it}}$ onward, all subsequent iterations corresponded to steady-state behavior. DoA streams generated with model I were obtained by setting σ=10°, streams generated with model II were obtained by setting $\sigma_{k}∼L\left(1.9{}^{\circ},0.6{}^{\circ}\right)$, and those generated with model III were obtained by using the same $\sigma_{k}$ distribution and setting $\nu_{k}∼G\left(\theta_{k}^{\;\mathrm{GT}\;},0.8\right)$. We verify that DoA measurement biases negatively impact the accuracy of the proposed method. More interestingly, we observe that the error-based policies (i.e., the ones using either the error or the cost as penalty factors) have higher accuracy when the DoA measurements on each array are unbiased (i.e., for DoA stream models I and II). This is to be expected since these policies penalize arrays with higher costs, and for unbiased DoA streams, higher costs are associated with less accurate estimates of the sound source position. However, this is no longer the case for DoA streams generated according to model III, where arrays are characterized by biased DoA estimates. Indeed, the local costs of each array may have minima at positions far from each other and from the actual source position $s^{\mathrm{GT}\;}$. In this scenario, the adaptive distance policy shows better accuracy, as distant arrays have on average the worst estimates of DoA and thus of source position. Therefore, depending on the specific DoA estimation procedure, which in turn leads to different DoA streams, it may be appropriate to choose the combination policy for diffusion-based SSL accordingly.

### 5.2. Acoustic Simulations

We now evaluate the localization performance of diffusion-based SSL in a simulated reverberant environment. To simulate the acoustic environment, microphone signals are generated using the image source method [42]. In particular, this method is used to generate all room impulse responses (RIRs) from all the candidate source positions in the 3D grid to all microphones. Then, we convolve the sound source signal with the simulated RIRs and corrupt the resulting signals with an additive Gaussian white noise with a signal-to-noise ratio (SNR) of 20 dB to obtain the simulated microphone signals. As a sound source, we used a 30-second male speech from the TIMIT database with a sampling frequency of Fs=16 kHz. It is also assumed that the sound source is omnidirectional.

Let us again consider K=8 uniform linear arrays (ULAs) of microphones whose reference points and orientations are set again according to Table 1. Also, each array is composed of $N_{\mathrm{mic}}=6$ elements with an inter-element distance of δ=2 cm. Therefore, the 3D coordinates of the *n*th microphone in array *k* can be defined as

(30) $$m_{k,n}=m_{k}+v_{k}\left(\delta \left(n-\frac{N_{\mathrm{mic}}+1}{2}\right)\right)$$

where $m_{k,n}$ denotes the *n*th microphone position within the *k*th array, and $n=1,\ldots ,N_{\mathrm{mic}}$. To obtain a DoA stream, we divide the simulated microphone signals into frames of length 2048 samples, each of which represents the data measurement at iteration step *i*. In each frame, microphone arrays estimate an “instantaneous” DoA using a beamforming technique. Specifically, we transform the received microphone signals into the time–frequency domain using the short-time Fourier transform (STFT) with a Hamming analysis window of length 256 samples overlapped by 50%. This results in a total of W=15 time windows per frame.

Let $X_{k,n}\left(t_{p},\omega_{q}\right)$ denote the STFT of the *n*th microphone signal of the *k*th array, evaluated at a time–frequency bin $\left(t_{p},\omega_{q}\right)$, where the index $t_{p}$ refers to the *p*th time segment, and $\omega_{q}$ refers to the *q*th frequency bin. Note that the STFTs are calculated at each iteration index *i*, but we omit this dependency to simplify the notation. The minimum variance distortionless response (MVDR) pseudospectrum at $\omega_{q}$ is given by [43]

(31) $$H_{k}\left(\theta_{r},\omega_{q}\right)=\frac{1}{a^{T}\left(\theta_{r},\omega_{q}\right)G_{k}^{-1}\left(\omega_{q}\right)a\left(\theta_{r},\omega_{q}\right)},$$

where $a(\theta_{r},\omega_{q})$ is the far-field propagation vector for each microphone array, computed for a set of sampled angles $\theta_{r}$, in radians, providing the desired angular resolution. The elements of the propagation vector $a(\theta_{r},\omega_{q})$ are given by

(32) $${\left[a(\theta_{r},\omega_{q})\right]}_{n}=e^{j\frac{\omega_{q}}{c}\delta \left(n-\frac{N_{\mathrm{mic}}+1}{2}\right)\cos\left(\theta_{r}\right)}$$

On the other hand, $G_{k}\left(\omega_{q}\right)$ in Equation (31) represents the sample estimate of the array covariance matrix, defined as

(33) $$G_{k}\left(\omega_{q}\right)=\frac{1}{W}\sum_{p=1}^{W}x_{k}\left(t_{p},\omega_{q}\right)x_{k}^{H}\left(t_{p},\omega_{q}\right).$$

where $x_{k}\left(t_{p},\omega_{q}\right)=\left[X_{k,1}\left(t_{p},\omega_{q}\right),\ldots ,X_{k,N_{\mathrm{mic}}}\left(t_{p},\omega_{q}\right)\right]$. Then, the DoA estimated by agent *k* at iteration index *i* is

(34) $$\theta_{k,i}=\arg\max_{\theta_{r}}h_{k}\left(\theta_{r}\right),$$

where $h_{k}\left(\theta_{r}\right)$ is the geometric mean of the pseudospectrum values $H_{k}\left(\theta_{r},\omega_{q}\right)$ along the frequency axis, with frequency bins in the range [500Hz,4kHz].

**Figure 5:**
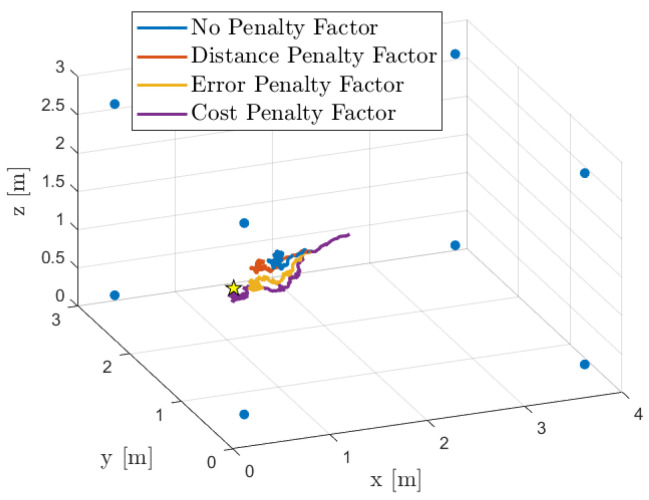
Simulated acoustic environment and localization results. The actual source position (depicted as a star) is at $s={\left[0.45,0.83,1.50\right]}^{T}$ m, and microphone array centers are shown as filled circles. The trajectory of estimated source locations, obtained using our diffusion-based SSL algorithm, illustrates the localization process.

We now present the localization results obtained from this acoustic simulation. First, as an illustrative example, Figure 5 shows the room layout (4 m × 3 m × 3 m), the positions of the eight microphone arrays (as listed in Table 1), and the true as well as estimated source locations. In this instance, the source is positioned at $s={\left[0.45,0.83,1.50\right]}^{T}$ m. Additionally, we illustrate the trajectories—the location estimates at each iteration—obtained using our diffusion-based SSL algorithm with all proposed combination policies.

**Figure 6:**
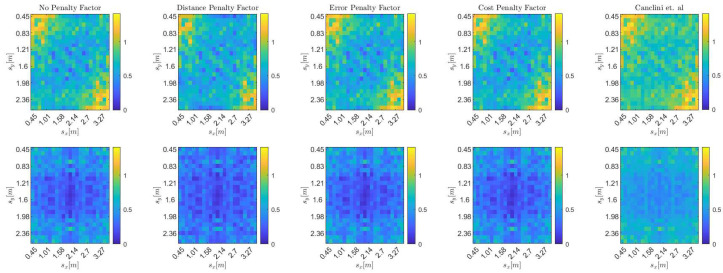
${\hat{\mathrm{MAE}}}_{\mathrm{avg}}$ at each candidate source position in a 23 × 23 grid on the xy plane for z=0.45 (**top**) and z=1.5 (**bottom**). Each column represents a different combination strategy, with the exception of the last column, which refers to the technique of Canclini et al. [24].

Further, following the methodology in the previous subsection, we evaluate localization accuracy using the average mean absolute error (${\hat{\mathrm{MAE}\;}}_{\mathrm{avg}}$), computed over the same 3D grid. However, in this case, we consider only a single realization, corresponding to the DoA stream obtained from the simulated acoustic environment. The results are shown in Figure 6, where the considered diffusion SSL and the method of [24] are compared. As we can see, the proposed approach always achieves higher accuracy than the centralized approach of [24], since the adaptive policies aim to penalize the arrays with the most harmful effects on localization. Moreover, the combination policies always improve the results compared to the trivial ATC diffusion implementation where no combination policies are used.

We can note that the localization performance obtained by using the combination policy based on the error-based penalty factor is similar to that of the combination policy based on the distance penalty factor. The reason is that in this simulated acoustic environment, and with the chosen DoA estimator, unlike the simpler DoA stream models of the previous subsection, while the bias increases, the DoA variance increases as well.

**Figure 7:**
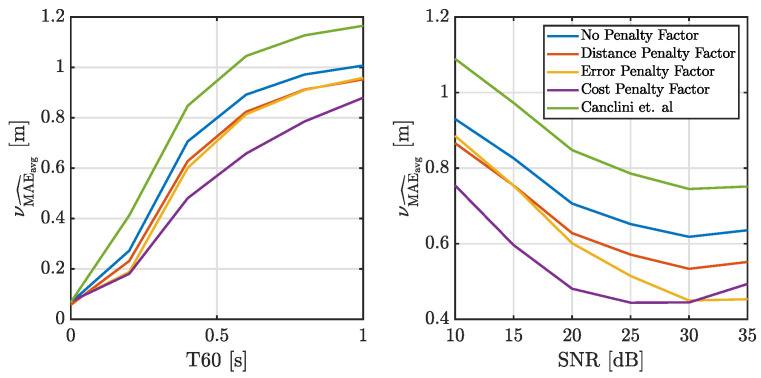
Average localization accuracy $\nu_{{\hat{\mathrm{MAE}}}_{\mathrm{avg}}}$ across the entire simulated acoustic environment (as described in Section 5.1), comparing the proposed method with various combination policies and the baseline method from Ref. Canclini. On the left, $\nu_{{\hat{\mathrm{MAE}}}_{\mathrm{avg}}}$ is obtained with T60 ranging from 0 to 1 s with a fixed SNR of 20 dB, while on the right, $\nu_{{\hat{\mathrm{MAE}}}_{\mathrm{avg}}}$ is obtained with a prescribed SNR ranging from 10 to 35 dB and a fixed T60 of 0.4 s. et al. [24].

We are interested in evaluating the robustness of the proposed method in adverse acoustic scenarios by assessing the localization accuracy when both T60 and SNR increase. Specifically, we measure the localization accuracy for different T60 values between 0 and 1 s at a fixed SNR of 20 dB. We also measure localization accuracy for a T60 of 0.4 s and varying SNR values between 10 and 35 dB. Instead of an analysis point by point, in this set of experiments we measure the average MAE over all the source positions in a subsampled grid of 5×5×3 points in the room, in order to have a mean volumetric localization accuracy value for each T60/SNR pair. In line with previous experiments, we assess the localization accuracy of every source location in the grid using the metric ${\hat{\mathrm{MAE}}}_{\mathrm{avg}}$, with $N_{\mathrm{it}}=100$. This metric is then averaged across the grid to obtain the mean volumetric average of localization accuracy, denoted as $\nu_{{\hat{\mathrm{MAE}}}_{\mathrm{avg}}}$. These results are summarized in Figure 7.

As expected, the performance of all methods deteriorates with increasing T60 and decreasing SNR. Nevertheless, even under the worst conditions, the diffusion-based methods outperform the centralized method proposed in [24]. We also observe that all the proposed adaptive combination policies result in improved accuracy and stability. Notably, the policy based on the cost penalty factor consistently delivers the best performance in terms of MAE.

## 6. A Study on Convergence and Stability Behavior

We now discuss the network dynamics of the proposed method, both in terms of convergence rate and steady-state stability. To asses these properties, we use the “instantaneous” MSD in Equation (24) as a metric, and evaluate it across several iterations. The network limit point is estimated according to Equation (25) with $N_{\mathrm{it}}=100$. Both analyses consider, without loss of generality, a single source position $s={\left[1.22,0.97,0.97\right]}^{T}$, generating a stream of DoAs according to model III (Section 5.1), and are averaged across 100 DoA stream realizations.

### 6.1. Convergence Speed

**Figure 8:**
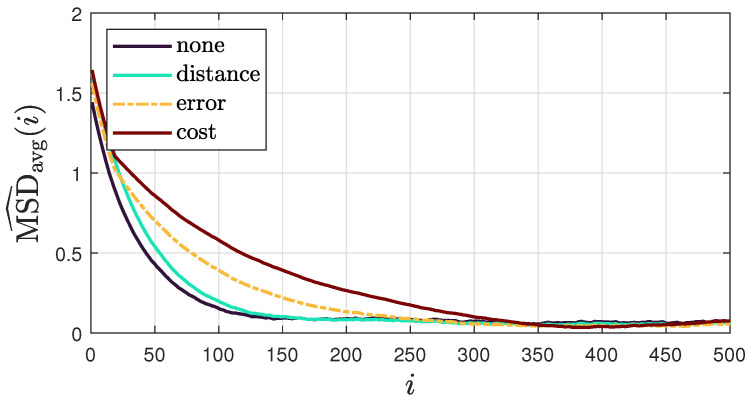
Average “instantaneous” MSD for each combination policy across 100 realizations of DoA streams, obtained using model III and considering a sound source of $s={\left[1.22,0.97,0.97\right]}^{T}$.

To evaluate the convergence speed of the proposed method, Figure 8 shows the average “instantaneous” MSD for each combination policy. The results show that combination strategies generally slow down convergence compared to scenarios with uniform weights (no combination policy). In particular, the cost policy significantly reduces convergence rates. This could be explained by the similarity between the cost policy and the adaptive relative variance policy, which is known for its slow convergence [44]. We also observe that among the combination policies, the distance-based policy achieves the fastest convergence rate, closely matching the rate observed when no combination policy is applied. This makes the distance-based policy preferable in scenarios where faster convergence is a priority.

**Figure 9:**
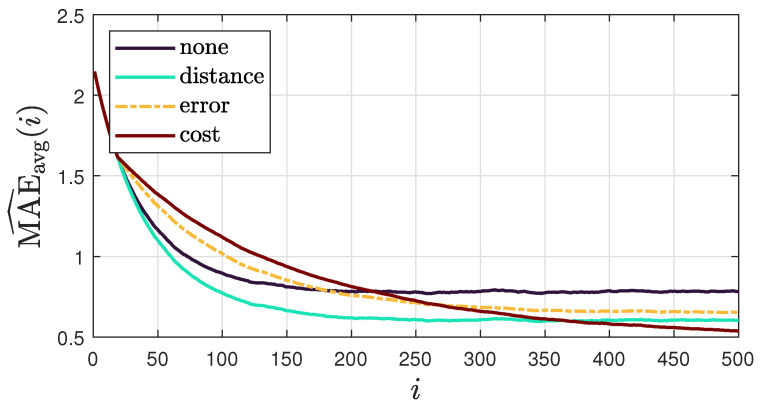
Average MAE for each combination policy across 100 realizations of DoA streams, obtained using model III and considering a sound source in $s={\left[1.22,0.97,0.97\right]}^{T}$.

We also analyze the evolution of the localization accuracy at each time-step by presenting the “instantaneous” network MAE in Figure 9, defined as ${\hat{\mathrm{MAE}}}_{\mathrm{avg}}\left(i\right)=\frac{1}{K}\sum {k=1}^{K}∥s_{k,i}-s^{\mathrm{GT}}∥$, to complement Figure 8. These results indicate that while some combination policies achieve faster convergence, they have minimal impact on overall localization accuracy. In particular, the distance-based policy, though slightly less accurate in the long run, provides a substantial improvement in convergence speed with only a minor trade-off in accuracy.

### 6.2. Steady-State Stability

We now examine how the step size $\mu_{k}$ influences the steady-state stability of the proposed ATC diffusion SSL. In the literature on diffusion networks, fixed step sizes $\mu_{k}$ are typically used [30,39,44,45]. Although they slow down convergence rates compared to decaying step sizes, they allow for adaptation to drifts in the data collected by the sensor nodes and are therefore often preferred [39]. Moreover, when both the local and global costs are convex, it is known that the step size can control the stability of the network solution at steady state. Recent studies have also confirmed this finding in non-convex environments [45], such as the ones commonly encountered in acoustic SSL, including the proposed approach.

**Figure 10:**
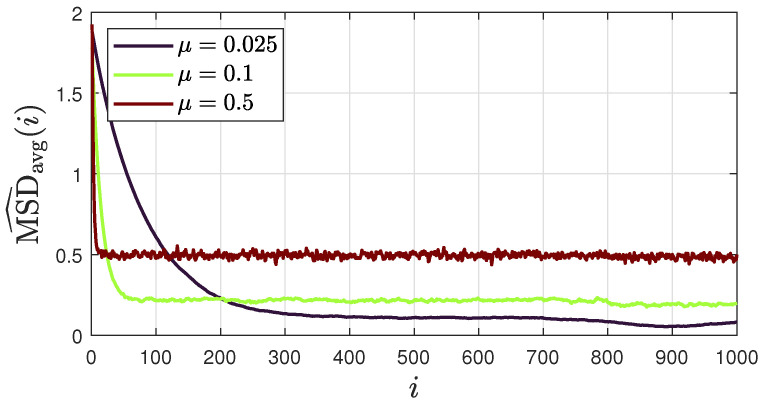
Average “instantaneous” MSD for different values of μ, across 100 realizations of DoA streams, obtained using model III and considering a sound source in $s={\left[1.22,0.97,0.97\right]}^{T}$. The results show the MSD performance if the distance combination policy is used.

Figure 10 shows the average “instantaneous” MSD for three different values of μ, namely, 0.25, 0.1, and 0.5. The results show that reducing the step size reduces the oscillations around the limit point at steady state, but at the cost of a slower convergence rate. This observation applies to all combination strategies. We therefore restrict ourselves to the distance combination policy, although similar behavior can be observed for other strategies as well.

These results highlight the trade-off between convergence speed and steady-state stability across different combination policies and step-size choices. Although formal convergence guarantees in this highly non-convex scenario remain challenging, extensive tests with varying source positions, room dimensions, and DoA stream statistics have shown that the proposed ATC diffusion SSL algorithm consistently converges to a stable solution. Notably, no instances of divergence were observed.

## 7. Impact of Network Connectivity on Localization Accuracy

**Figure 11:**
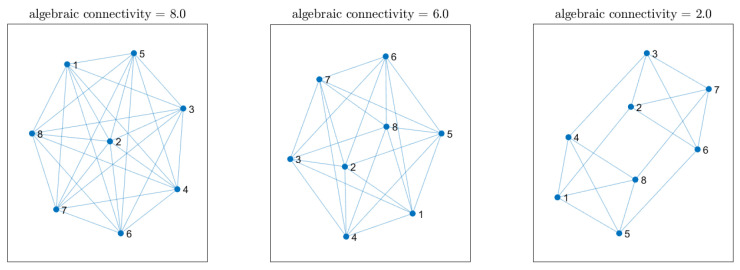
Different network topologies and their relative algebraic connectivity used to study performance as a function of connectivity.

**Figure 12:**
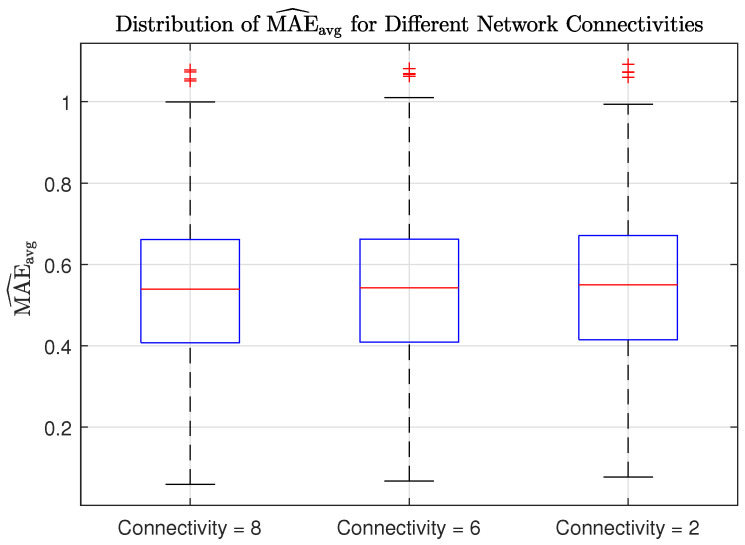
Distribution of ${\hat{\mathrm{MAE}}}_{\mathrm{avg}}$ for different network connectivities.

We now investigate how the localization performance is influenced by reduced network connectivity. Specifically, we modify the network by reducing the number of neighbors that each node has based on their Euclidean distance. In other words, if the distance between nodes (k,l) exceeds a certain threshold, both combination weights $w_{k,l}$ and $w_{l,k}$ are set to zero. Starting from the same array network configuration as in Section 5.2, we can find three distinct network topologies by gradually decreasing this distance threshold before the network splits into two separate subnetworks. These topologies are depicted in Figure 11, where self-loops have been omitted to avoid clutter. Their degree of connectivity is quantified by the well-known algebraic connectivity, which is defined as the second smallest eigenvalue of the Laplacian of the combination matrix W [46]. To assess the overall localization accuracy across different network connectivities, we computed the average MAE as described in the previous section, further averaging the error over every point in a 23×23×3 grid of possible source locations. For this experiment, we applied the cost penalty factor. Figure 12 shows the distribution of MAE values for each network connectivity. The results show that the localization accuracy is not significantly affected by sparser topologies, highlighting the effectiveness of the proposed method. Interestingly, the fully connected networked topology serves as the performance benchmark as it exhibits the highest accuracy.

## 8. Conclusions and Future Work

In this work, we reformulated the general problem of sound source localization for a network of acoustic arrays as a distributed optimization problem, where the arrays measure streams of acoustic parameters and cooperate to localize a sound source. In particular, we proposed ATC diffusion as a technique for localizing acoustic sources through cooperation between microphone arrays. We also discussed how localization performance can be improved by using different weighting schemes for communication between arrays, which we call combination policies.

As an example, we presented an ATC diffusion-based SSL method that enables 3D localization of a single sound source using 2D direction-of-arrival (DoA) measurements obtained from spatially distributed linear microphone arrays. This approach extends the work in [38]. Ad hoc combination policies were developed to improve localization accuracy, and all demonstrated superior performance compared to uniform combination policies, where communication links between agents are uniformly weighted. These results hold true for both statistical DoA stream models and simulated acoustic environments. We also showed that stability and convergence properties of the proposed approach can be controlled by the step-size parameters.

Future work will address more complex scenarios with multiple sound sources and different cost functions based on other sound propagation models and acoustic parameters, e.g., TDOAs, acoustic energy, and sound intensity.

## Figures and Tables

**Table 1:** Reference points and orientations of microphone arrays used in Section 5.

ID	$m_{k}$ [m]	$\left(\alpha_{k},\beta_{k}\right)$
1	${\left[0.25,0.25,0.25\right]}^{T}$	(−135°, 45°)
2	${\left[3.75,0.25,0.25\right]}^{T}$	(45°, 45°)
3	${\left[3.75,2.75,0.25\right]}^{T}$	(45°, 0°)
4	${\left[0.25,2.75,0.25\right]}^{T}$	(225°, 0°)
5	${\left[0.25,0.25,2.75\right]}^{T}$	(−45°, 0°)
6	${\left[3.75,0.25,2.75\right]}^{T}$	(135°, 45°)
7	${\left[3.75,2.75,2.75\right]}^{T}$	(135°, 0°)
8	${\left[0.25,2.75,2.75\right]}^{T}$	(45°, 45°)

## Data Availability

The data presented in this study are available on request from the corresponding author.
